# Interregional correlations of glucose metabolism between the basal ganglia and different cortical areas: an ultra-high resolution PET/MRI fusion study using ^18^F-FDG

**DOI:** 10.1590/1414-431X20176724

**Published:** 2017-11-13

**Authors:** J.H. Kim, Y.D. Son, J.M. Kim, H.K. Kim, Y.B. Kim, C. Lee, C.H. Oh

**Affiliations:** 1 Research Institute for Advanced Industrial Technology, College of Science and Technology, Korea University, Sejong, South Korea; 2 Department of Biomedical Engineering, College of Health Science, Gachon University, Incheon, South Korea; 3 Department of Electronics and Information Engineering, College of Science and Technology, Korea University, Sejong, South Korea; 4 Department of Neurosurgery, Gachon University Gil Medical Center, Gachon University School of Medicine, Incheon, South Korea; 5 Bioimaging Research Team, Korea Basic Science Institute, Cheongju, South Korea

**Keywords:** Basal ganglia, ^18^F-FDG PET/MRI, ROI-based interregional correlation analysis, Functional correlation, Glucose metabolism

## Abstract

Basal ganglia have complex functional connections with the cerebral cortex and are involved in motor control, executive functions of the forebrain, such as the planning of movement, and cognitive behaviors based on their connections. The aim of this study was to provide detailed functional correlation patterns between the basal ganglia and cerebral cortex by conducting an interregional correlation analysis of the ^18^F-fluorodeoxyglucose (^18^F-FDG) positron emission tomography (PET) data based on precise structural information. Fifteen participants were scanned with 7-Tesla magnetic resonance imaging (MRI) and high resolution research tomography (HRRT)-PET fusion system using ^18^F-FDG. For detailed interregional correlation analysis, 24 subregions of the basal ganglia including pre-commissural dorsal caudate, post-commissural caudate, pre-commissural dorsal putamen, post-commissural putamen, internal globus pallidus, and external globus pallidus and 80 cerebral regions were selected as regions of interest on the MRI image and their glucose metabolism were calculated from the PET images. Pearson's product-moment correlation analysis was conducted for the interregional correlation analysis of the basal ganglia. Functional correlation patterns between the basal ganglia and cerebral cortex were not only consistent with the findings of previous studies, but also showed new functional correlation between the dorsal striatum (i.e., caudate nucleus and putamen) and insula. In this study, we established the detailed basal ganglia subregional functional correlation patterns using ^18^F-FDG PET/MRI fusion imaging. Our methods and results could potentially be an important resource for investigating basal ganglia dysfunction as well as for conducting functional studies in the context of movement and psychiatric disorders.

## Introduction

The basal ganglia are a group of subcortical gray nuclei, which include the dorsal striatum [i.e., caudate nucleus (CAU) and putamen (PUT)], ventral striatum [including the nucleus accumbens (NA)], globus pallidus (GP), substantia nigra (SN), and subthalamic nucleus (STN), and have complex anatomical and functional projections in the brain (1,2). In these projections, the CAU, PUT, and NA serve as the main cortical inputs, and the internal globus pallidus (GPi) and SN pars reticulata (SNr) serve as the main cortical outputs (1). Regarding functional connections of the basal ganglia, some previous studies have suggested the existence of three segregated parallel functional projection models such as motor, association, and limbic loop models (1,2). According to these models, each striatal region receives its inputs from the different cortical areas and projects back to the same cortical regions via the thalamus (THA) (1,2). Based on their connections, the basal ganglia are involved in motor control, cognition function, and emotional and motivational processes (1,2). However, these models were based on anatomical information in nonhuman primate and human *in vivo*. For better understanding of the basal ganglia function, anatomical information is also necessary, but does not provide functional information directly. Therefore, we hoped to investigate detailed functional correlation patterns between the basal ganglia and different cortical areas in the resting state using magnetic resonance imaging (MRI) and positron emission tomography (PET) with ^18^F-fluorodeoxyglucose (^18^F-FDG).

A brain-dedicated high resolution research tomograph (HRRT)-PET/7-Tesla MRI fusion system is one of the most attractive imaging tools for functional studies on the basal ganglia, because the basal ganglia require high spatial resolution to observe their subregions and diverse functions. In addition, because the PET/MRI fusion system could provide ^18^F-FDG PET images that are spatially matched with 7-Tesla MRI images, it is possible to investigate the functional activity of the basal ganglia and other brain regions without any post-processing of PET and MRI data. Several PET/MRI studies have demonstrated that the PET/MRI fusion system could differentiate between small brain structures *in vivo*, such as the hippocampus, THA, brainstem nuclei, and SN, and determine their corresponding molecular information (3,4). Therefore, we used this imaging fusion system to measure the functional activity of the basal ganglia and cortical areas in the human brain *in vivo*.

## Material and Methods

### Healthy subjects

The study protocol was approved by the Institutional Review Board of the Gachon University Gil Medical Center (Incheon, South Korea), and was performed in accordance with the Declaration of Helsinki. Fifteen healthy controls (13 men and 2 women) were recruited from the Gachon University Gil Medical Center. The criteria for participating in the study were as follows: 1) age between 20 and 30 years; 2) absence of current or past psychiatric, neurological, or medical illness, and 3) absence of current use of any medication. Before the PET scans, all subjects underwent urine tests to exclude substance abuse and pregnancy in the participants. None of the volunteers had any abnormalities of the gross brain structures visualized with MRI.

### Scan protocol

All subjects were scanned using a brain-dedicated PET/MRI fusion system (Neuroscience Research Institute, Gachon University, Incheon, South Korea) developed by combining HRRT-PET (Siemens, USA) and 7-Tesla MRI (Siemens, Germany) with a shuttle bed (5). For all subjects, a bolus injection of ^18^F-FDG (213.3±41.6 MBq; range=153.5–302.3 MBq) was administered intravenously. For the uptake of ^18^F-FDG into the brain, the subjects lay on a bed in a dark room with their eyes closed for 30 min. After ^18^F-FDG uptake, an emission scan was performed in the static mode for 30 min. For attenuation correction of the emission scan, a transmission scan was conducted for 6 min using a 137Cs point source. After the PET scans, a 7-Tesla MRI scan was performed for structural brain imaging by using a three-dimensional (3-D) T1-weighted magnetization-prepared and rapid gradient-echo (T1-MPRAGE) sequence. The 3-D T1-MPRAGE images were acquired with the following parameters: repetition time=1,900 ms, echo time=3.73 ms, inversion time=1,100 ms, flip angle=10°, voxel size=0.8×0.8×0.8 mm^3^, and number of slices=256.

The ^18^F-FDG PET images were reconstructed with a 3-D ordinary Poisson ordered-subset expectation maximization (OP-OSEM) algorithm that was based on the symmetry and single-instruction multiple-data (SIMD)-based projection and back-projection (6,7). The reconstructed PET images were performed with a decay correction, and they had 256×256×207 matrix and 1.22×1.22×1.22 mm^3^ voxel resolution. For calculation of the ^18^F-FDG standardized uptake value ratio (SUVR), the ^18^F-FDG PET emission data were reconstructed as a single frame. The reconstructed PET and MRI data were spatially coregistered on the basis of the calibrated coordinates without additional data processing, similar to PET/CT (5).

### Image analysis

**Figure 1.**
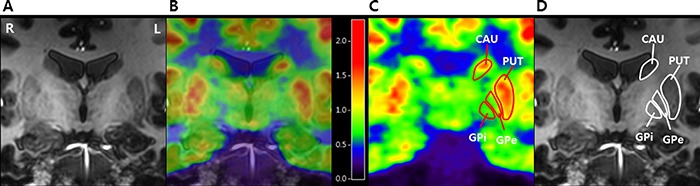
A 7-Tesla MRI image of the GPi and GPe (*A*), a^18^F-FDG PET/MRI fusion image (*B*), a^18^F-FDG HRRT-PET image with labeling (*C*), and selected regions of interest and labels that were based on the 7-Tesla MRI image (*D*). CAU: caudate nucleus; GPe: external globus pallidus; GPi: internal globus pallidus; L: left; PUT: putamen; R: right.

The ^18^F-FDG SUVR images were acquired by normalizing the uptake value of the whole brain to the uptake value of the cerebellum from the ^18^F-FDG PET data. As shown in Figure 1, the anatomical locations of the GPi and external globus pallidus (GPe) were differentiated with the PET/MRI fusion images by referring to major landmarks, including the CAU, PUT, THA, and red nucleus according to an anatomical reference (8). Based on such anatomical information, the regions of interest (ROIs) of the bilateral GPi and GPe were manually drawn on each MRI image with VINCI software (Max Planck Institute for Metabolism Research, Germany). The identifiability of the bilateral GPi and GPe regions was rated by two trained researchers (KJH and KJM). In addition, the ^18^F-FDG SUVR values were obtained from individual ^18^F-FDG PET images corresponding with the 3-D T1-MPRAGE images.

Spatial normalization of both ^18^F-FDG SUVR and 7-Tesla MRI images was conducted with statistical parametric mapping 8 (SPM8) software (Wellcome Trust Center for Neuroimaging, University College London, England) in order to acquire the ^18^F-FDG SUVR values of all brain structures, excluding the cerebellum. The ^18^F-FDG SUVR values were obtained in 116 predefined ROIs with an automated anatomical labeling program (9). Additionally, the bilateral pre-commissural dorsal caudate (PreDCAU), post-commissural caudate (PostCAU), pre-commissural dorsal putamen (PreDPUT), post-commissural putamen (PostPUT), NA, SN, and STN were selected as the ROIs. These ROIs were drawn on spatially normalized 7-Tesla MRI images according to an anatomical reference (8), and their ^18^F-FDG SUVR values were obtained with the spatially normalized ^18^F-FDG PET images. In the process of obtaining the ^18^F-FDG SUVR value of the ROIs for interregional correlation analysis, we assumed that brain regions whose ^18^F-FDG SVUR values were significantly associated to be functionally correlated; therefore, when ^18^F-FDG SVUR value alters in a single brain region, the alteration will affect the ^18^F-FDG SVUR values of other brain areas with which it intercorrelates.

### Statistical analysis

Statistical analyses were conducted with the SPSS statistical software package (version 21; IBM Corporation, USA). As mentioned above, the number of male participants was greater than that of female participants. In order to avoid any possible confounding effects, gender was used as a covariant in the statistical methods, where appropriate.

Based on some previous studies suggesting that the strength of functional correlation between brain regions is proportional to the magnitude of the correlation coefficient (10–12), the ROI-based interregional correlation analyses were performed between the ^18^F-FDG SUVR values of the basal ganglia and related brain regions using Pearson's product-moment correlation. The level of statistical significance was defined as P*<*0.05 (two-tailed). In addition, an interrater reliability on the GPi and GPe regions was assessed using interclass correlation coefficient. The level of statistical significance was defined as P*<*0.001 (two-tailed).

## Results

### ROI-based interregional correlation patterns between the 18F-FDG SUVR values in the basal ganglia and cortical areas

The bilateral CAU and PUT, parts of the dorsal striatum, showed widespread positive correlations with entire cortical areas. Left CAU had strong positive correlations with the right rolandic operculum, left precuneus (PCUN), GP, and THA, and bilateral frontal and temporal lobes, superior and middle occipital gyri (SOG and MOG), limbic lobe, insula (INS), amygdala (AMY), and PUT. Right CAU had strong positive correlations with the right postcentral gyrus (POST), anterior cingulate gyrus (ACG), hippocampus (HIP), and INS, left THA, and bilateral frontal lobe, angular gyrus (AG), supramarginal gyrus (SMG), occipital lobe, and PUT. Left PUT was strongly associated with the right precentral gyrus (PRE), POST, and SMG, left inferior temporal gyrus (ITG), AG, GP, and THA, and bilateral frontal, occipital, and limbic lobes, PCUN, INS, and CAU. Right PUT was strongly correlated with the right HIP and AMY, left GP and THA, and bilateral frontal and occipital lobes, INS, CAU, SN, and STN.

The bilateral PreDCAU, PostCAU, PreDPUT, and PostPUT showed widespread positive correlations with entire cortical areas. Left PreDCAU was strongly correlated with the right SMG, left GP, and bilateral PRE, POST, frontal lobe, PCUN, and median and posterior cingulate gyri (MCG and PCG). Right PreDCAU was strongly associated with the right superior frontal gyrus (medial orbital; MOrG), SMG, GPi, and STN, left PCUN and GPe, and bilateral occipital lobe, MCG, PCG, and GP. Bilateral PostCAU had strong correlations with the right INS, left THA, and bilateral PRE, POST, and frontal, parietal, and limbic lobes. Left PreDPUT was strongly correlated with the right PCG, SN, and STN, left olfactory cortex (OLF), MCG, and GP, and bilateral NA, and right PreDPUT was strongly associated with the right PCG, left MOG, and bilateral NA. Bilateral PostPUT had strong correlations with the right GPi, left GP, and bilateral MCG, GPe, and STN. The bilateral NA showed positive correlations with frontal, temporal, occipital, and limbic lobes, INS, and subcortical gray nuclei. Particularly, left NA showed strong correlations with the right PCG and left OLF, and right NA showed strong associations with the right PCG and left superior frontal gyrus (orbital part; SFGOr), OLF, and MOG.

Like the dorsal striatum (i.e., CAU and PUT), the bilateral GP showed widespread positive correlations with entire cortical areas. Especially, left GP showed strong correlations with the right HIP and GPi, left CAU and NA, and bilateral frontal lobe, PCUN, SOG, MOG, Cuneus (CUN), MCG, PCG, INS, PUT, GPe, and STN, and right GP showed strong associations with the right SOG and GPi, left AG, and bilateral MOG, CUN, MCG, and STN. In addition, the bilateral GPi showed widespread positive correlations with cortical areas excluding temporal lobe. Specifically, left GPi showed strong correlations with the right supplementary motor area (SMA), left PCUN, and bilateral cingulate gyri and GPe, and right GPi showed strong associations with the right superior and middle frontal gyri (SFG and MFG), left PRE, POST, superior frontal gyrus (medial; SFGM), AG, GP, and STN, and bilateral PCUN, MCG, PCG, and GPe. Like the GPi, the bilateral GPe showed widespread positive correlations with cortical areas excluding temporal lobe. Particularly, left GPe was strongly correlated with the right SOG, ACG, and MCG, left PCG and GP, and bilateral GPi, and right GPe was strongly associated with the left PCUN and GP and bilateral MCG, PCG, and GPi.

The bilateral SN showed widespread positive correlations with entire cortical areas excluding central region and had particularly strong correlations with the right HIP and bilateral SOG, CUN, and STN.

Lastly, the bilateral STN showed widespread positive correlations with entire cortical areas excluding temporal lobe and INS. Especially, left STN was strongly correlated with the right MOG, CUN, PUT, and GPi and bilateral AG, SOG, GP, and SN, and right STN was strongly associated with the right SOG, left MCG and parahippocampal gyrus (PHG), and bilateral GP and SN.

**Figure 2.**
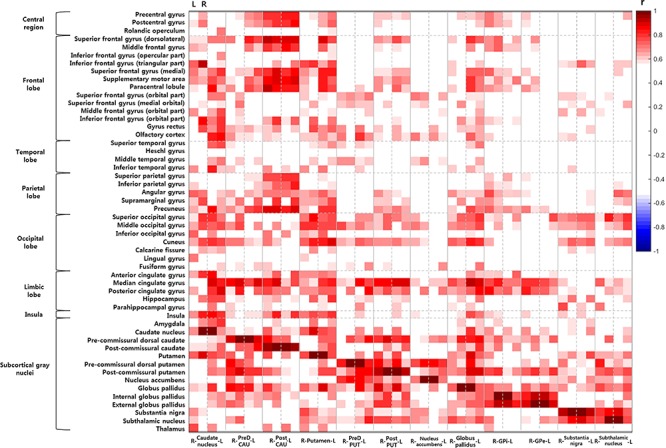
ROI-based interregional correlation matrix of the basal ganglia and related brain structures. The colors represent the significance of the correlational coefficients. GPe: external globus pallidus; GPi: internal globus pallidus; L: left; PostCAU: post-commissural caudate; PostPUT: post-commissural putamen; PreDCAU: pre-commissural dorsal caudate; PreDPUT: pre-commissural dorsal putamen; R: right; r: correlation coefficient; ROI: region of interest. Pearson's product-moment correlation analysis was used for statistical analysis. The level of statistical significance was defined as P*<*0.05 (two-tailed).

The results of the ROI-based interregional correlation analysis are shown in Figure 2.

### Interrater reliability of glucose metabolism in the GPi and GPe

**Figure 3.**
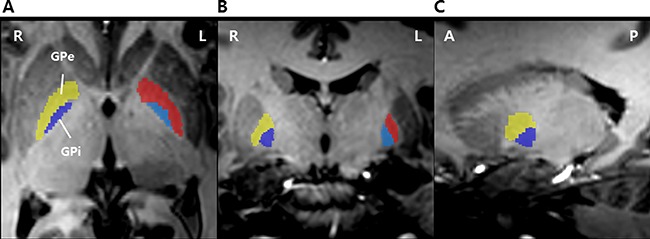
Volume segmentation of the GPi and GPe in the 7-Tesla MRI image. Volume segmentation of the GPi and GPe on the transaxial (*A*), coronal (*B*), and sagittal planes (C). Yellow overlay: manual labeling of the right GPe; red overlay: manual labeling of the left GPe; navy overlay: manual labeling of the right GPi; and sky-blue overlay: manual labeling of the left GPi. A: anterior; GPe: external globus pallidus; GPi: internal globus pallidus; L: left; P: posterior; R: right.

To assess the interrater reliability of glucose metabolism in the GPi and GPe, volume segmentation of these regions was conducted with the 7-Tesla MRI images (Figure 3), and their glucose metabolism were measured in the ^18^F-FDG PET images. Here, the raters (KJH and KJM) who identified the ROIs underwent interrater reliability tests. The interclass correlation coefficient was very high (P*<*0.001), as shown in Table 1.

**Table 1.** ^18^F-FDG SUVR values in the internal and external globus pallidi and interrater reliability.

	GPi_L	GPi_R	GPe_L	GPe_R
**Rater 1**				
Mean	0.738	0.749	0.829	0.843
Standard deviation	0.075	0.075	0.067	0.068
Minimum	0.627	0.625	0.739	0.743
Maximum	0.887	0.891	0.955	0.949
**Rater 2**				
Mean	0.748	0.758	0.851	0.845
Standard deviation	0.079	0.082	0.064	0.073
Minimum	0.632	0.614	0.737	0.732
Maximum	0.894	0.932	0.985	0.995
**Interrater reliability test**				
Interclass correlation coefficient	0.980	0.947	0.960	0.980
(P value)	(<0.001)	(<0.001)	(<0.001)	(<0.001)

GPe_L: left external globus pallidus; GPe_R: right external globus pallidus; GPi_L: left internal globus pallidus; GPi_R: right internal globus pallidus; SUVR: standardized uptake value ratio. Interclass correlation coefficient was used for statistical analysis.

## Discussion

We conducted a detailed interregional correlation analysis of the glucose metabolism between the basal ganglia and different cortical areas in healthy subjects using an ultra-high resolution ^18^F-FDG PET/MRI fusion system in order to increase the understanding of the basal ganglia function.

In the present study, an ultra-high resolution ^18^F-FDG PET/MRI fusion imaging enabled a distinction between the GPi and GPe regions and quantitative evaluation of their glucose metabolism. In addition, the ROI-based correlation analysis of the regional glucose metabolism enabled to investigate the characteristic patterns of functional correlation between the basal ganglia and different cortical areas in the resting state. Several studies of ^18^F-FDG PET-based interregional correlation analyses have been conducted to observe the patterns of brain functional correlation and their results have suggested that the patterns of ^18^F-FDG PET-based interregional correlations might reflect functional and/or metabolic connection (13–15).

In these analyses, the subregions of the basal ganglia showed various functional correlation patterns in the whole brain. In the dorsal striatum, the PUT had mainly strong correlations with the primary motor cortex, SMA and ACG, involving in motor function (16). This is consistent with the idea that the PUT is a main structure in the motor system. Additionally, the dorsal striatum was strongly associated with dorsolateral prefrontal cortex (DLPFC) and ACG, involved in executive function (16). These correlations suggest that the dorsal striatum may be concerned in higher-level cognitive function based on their functional connection. Moreover, although we did not investigate several segregated thalamostriate connections with respect to anatomical locations of individual thalamic nuclei, our study found that the dorsal striatum is strongly associated with the THA, consistent with both motor and cognitive loop models (1,2). However, additional studies will be required in the future for better observation of these connections. The dorsal striatum also showed strong correlations with the insula. The insula is anatomically interconnected with various structures including brainstem nuclei, limbic structures, THA, AMY, basal ganglia, and prefrontal cortex, and plays a role in processing gustatory, auditory, olfactory, language, pain, visceral motor/sensory, somatic sensation, and modulating attention and emotion based on their connections (17–20). The dorsal striatum is also related to most of these functions. Although the insula is not an interesting brain area in the segregated parallel loop models (1,2), this finding suggests that the insula is of considerable importance in functional loop models of the basal ganglia.

Some studies proposed three distinct striatal functional areas (1,2); associative striatum (consisting of the PreDCAU, PostCAU, and PreDPUT), sensorimotor striatum (consisting of the PostPUT), and limbic striatum (consisting of the NA). Our study investigated functional correlation patterns based on these functional areas. Here, it is notable that both the PreDCAU and PostCAU are strongly correlated with the frontal, parietal, occipital, and limbic lobes, whereas the PreDPUT is weakly associated with the temporal lobe. This is consistent with the concept that the associative striatum is functionally connected with the association cortices (1,2). Also notable is that the bilateral NA have positive correlations with right PCG. This is consistent with the concept that the NA is a main component of cognitive loop model (1,2). However, we did not find it to be significantly correlated with the sensorimotor striatum.

In the correlation analysis of the basal ganglia, we found that the GP was strongly correlated with both left CAU and bilateral PUT, consistent with previous proposed loop models (1,2). Although ultra-high resolution PET/MRI fusion imaging is sufficient to distinguish between the GPi and GPe regions, we could not find significant correlations between subdivisions of the GP and dorsal striatum. These findings suggest that this does not always lead to increases of the glucose metabolism in the subdivisions of the GP due to an inhibitory effect within the gamma-aminobutyric acid (GABA)ergic projection between subdivisions of the GP and dorsal striatum. We also found no significant correlations with the SN based on parallel loop models (1,2). This may be because we investigated significant functional associations between the basal ganglia and whole SN instead of the SNr, which is a region connected with the dorsal striatum, GPi, and GPe.

The STN is one of the main cortical inputs in anatomical projections (1) and is anatomically connected with primary motor area, GPe, THA, SN pars compacta, ventral tegmental area, and brainstem nuclei. Here, we did not find it to be significantly correlated with the STN based on their anatomical projections. This may be because functional correlations do not always depend on direct anatomical projections.

The present study had some limitations. The major limitation was the relatively small sample size (n=15). The sample size might not have provided sufficient power for the statistical analyses. However, the main objective of the present study was the demonstration of the feasibility for identifying the detailed characteristic patterns of the functional correlation between the basal ganglia and related brain structures. Another limitation was that only radiotracer ^18^F-FDG was used in this study. The basal ganglia connections include dopaminergic, glutamatergic, and GABAergic connections. Therefore, functional correlations between the basal ganglia and cortical areas should be further investigated with ultra-high resolution PET/MRI fusion imaging techniques using ^18^F-FDG as well as multiple tracers, in order to probe the pre- and post-synaptic markers of the dopaminergic, glutamatergic, and GABAergic systems.

Despite these limitations, we came to the following main conclusions based on the results: 1) the ROI-based interregional correlation analysis with ultra-high resolution ^18^F-FDG PET/MRI fusion imaging provided important functional correlation patterns in the subregions of the basal ganglia and related structures and 2) these imaging and analysis methods and our results could be an important tool and a resource for identifying the basal ganglia functional correlations as well as conducting the observation of abnormal functional correlations in clinical studies, such as movement and psychiatric disorders.
